# From anticipatory fear to relational trust: women's experiences of gynecological examination in Turkey: a qualitative study

**DOI:** 10.3389/frph.2026.1955456

**Published:** 2026-10-01

**Authors:** Çiğdem Can Bayrak, Ayse Sezer Balci

**Affiliations:** 1 Department of Obstetrics and Gynecology, Bucak State Hospital, Burdur, Türkiye; 2 Department of Public Health Nursing, Faculty of Health Sciences, Burdur Mehmet Akif Ersoy University, Burdur, Türkiye

**Keywords:** fear, gynecological examination, health communication, qualitative research, trust

## Abstract

**Background:**

While fear, embarrassment, and sociocultural barriers associated with gynecological examinations are well documented, existing literature has predominantly focused on their prevalence and consequences, with limited attention to the dynamic processes through which these meanings are constructed and transformed within the clinical encounter.

**Aim:**

This study aimed to explore how women construct, experience, and reinterpret gynecological examinations and to examine how communication and interpersonal interactions shape the negotiation of fear and trust during the clinical encounter.

**Methods:**

This qualitative study adopted a qualitative descriptive design informed by a social constructionist perspective. Twenty women who had undergone vaginal speculum examination in the outpatient gynecology clinic of a public hospital in Burdur, Turkey, were recruited using purposive sampling. Data were collected through semi-structured, in-depth interviews conducted immediately after the examination and analyzed using reflexive thematic analysis following Braun and Clarke's approach.

**Results:**

Four overarching themes and six interrelated subthemes were identified: (1) Constructing Fear Before the Examination, (2) Negotiating Embarrassment and Bodily Exposure, (3) Communication and the Development of Relational Trust, and (4) Reinterpreting the Examination Experience. Across these themes, anticipatory fear was often shaped by socially transmitted narratives and uncertainty, whereas communication and relational trust facilitated the reinterpretation of the examination as less distressing than anticipated.

**Conclusion:**

Gynecological examination should be understood not only as an invasive clinical procedure but also as a socially and relationally mediated experience. Strengthening patient-centered communication and privacy-sensitive care may improve women's engagement with gynecological services.

## Introduction

Gynecological examinations, including pelvic and vaginal speculum assessment, remain a fundamental component of women's healthcare, particularly in the context of cervical cancer screening and the evaluation of gynecological conditions. International guidelines emphasize the importance of early detection strategies, including regular screening, for reducing morbidity and mortality associated with cervical cancer (1).

Despite their critical role in preventive care, many women experience significant discomfort during gynecological procedures. This discomfort often stems from the intimate and invasive nature of pelvic and vaginal speculum examinations and may be accompanied by increased anxiety and embarrassment (2, 3). These negative experiences extend beyond immediate procedural reactions and have significant implications for health-related behaviors. In particular, fear and anxiety associated with gynecological examinations have been identified as key determinants of delayed attendance, reduced screening uptake, and, in some cases, complete avoidance of preventive services, thereby representing a substantial public health challenge (3).

While fear and avoidance related to gynecological examinations are well documented, existing research has largely focused on their prevalence and consequences, with limited attention to the dynamic processes through which these meanings are constructed and transformed within clinical encounters. Although international research has increasingly explored women's experiences of gynecological examination, qualitative evidence from Turkey remains limited and largely concentrated on specific contexts such as childbirth or general examination experiences, with relatively few studies focusing specifically on vaginal speculum examination from the patient perspective (4–6).

Women's fear of gynecological examination appears to be shaped more by socially constructed meanings than by direct experiential factors, highlighting the need to move beyond purely experience-based explanations (7). From a social constructionist perspective, meanings are produced through social interactions and culturally shared narratives (5).

Within this framework, clinical communication functions not merely as a supportive component of care but as a central relational mechanism through which these constructed meanings are negotiated and potentially transformed. Inadequate communication may reinforce vulnerability and fear, whereas clear, empathetic, and patient-centered communication can reduce uncertainty, restore a sense of control, and facilitate a shift toward trust. Thus, communication operates as a meaning-making process through which women interpret and reconstruct their examination experience (8–10). Despite its central role, the process through which communication transforms fear into trust remains insufficiently conceptualized within existing qualitative literature. Importantly, the novelty of the present study lies in examining these experiences as a dynamic process rather than as isolated emotional responses. By following women's accounts across the examination trajectory, this study illustrates how socially constructed anticipatory fear and vulnerability may be renegotiated through communication and relational trust and subsequently reinterpreted in light of the actual examination experience. Accordingly, this study aimed to explore how women construct and reinterpret their experiences of gynecological examination and to examine the role of communication and relational trust within this process.

This study aims to address the following research questions:

1) How are fears and expectations regarding gynecological examination socially constructed?
2) How do women negotiate vulnerability during the examination?
3) How do communication and interpersonal interactions shape women's experiences?
4) How do women reinterpret the examination after it is completed?

## Materials and methods

### Study design

This study adopted a qualitative descriptive design informed by a social constructionist perspective. The qualitative descriptive design was used to remain closely grounded in participants' accounts of their experiences of vaginal speculum examination, while the social constructionist perspective informed how these accounts were interpreted. From this perspective, meanings attributed to fear, embarrassment, vulnerability, communication, and trust were understood as shaped through social interactions, culturally shared expectations, and the clinical encounter rather than as fixed or purely individual experiences.

Reflexive thematic analysis, following Braun and Clarke (2022) was used to develop patterns of shared meaning across participants' accounts. The analytic process was therefore not understood as the discovery of pre-existing themes within the data; rather, themes were actively developed through the researchers' iterative engagement with the participants' accounts. The social constructionist perspective informed this interpretive process by directing attention to how participants constructed and negotiated meanings of the examination before, during, and after the clinical encounter. The researchers' interpretive role was considered part of knowledge production rather than an influence that could be fully separated from the analytic process (11).

The study was reported in accordance with the Consolidated Criteria for Reporting Qualitative Research (COREQ) checklist (12).

### Participants and procedures

The study was conducted in the outpatient gynecology clinic of a public hospital in Burdur, Turkey. Burdur is a medium-sized province located in southwestern Turkey, with a population of approximately 280,000 inhabitants. The local economy is primarily based on agriculture, animal husbandry, education, and public services. The study setting reflects a typical public healthcare environment serving both urban and rural populations.

Data collection took place between April 2026 and May 2026. All participants underwent the vaginal speculum examination performed by a female gynecologist. All interviews were conducted immediately after the examination to minimize recall bias and capture participants' immediate emotional and experiential responses.

Potential participants were identified by clinic staff following completion of their gynecological examination. Eligible women were approached by the researcher in a private area of the clinic, informed about the study, and invited to participate voluntarily. Purposive sampling was employed to ensure the inclusion of participants who had direct and recent experience of vaginal speculum examination, thereby enabling in-depth exploration of the phenomenon (13). Women were eligible for inclusion if they were 18 years of age or older, had undergone a vaginal speculum examination, could communicate in Turkish, and provided informed consent. Women experiencing severe emotional distress were excluded for ethical reasons, as participation immediately following the examination could increase emotional burden and compromise their ability to provide informed and voluntary responses.

In keeping with the flexible and iterative nature of reflexive thematic analysis, the final sample size was not determined through statistical considerations or a predetermined numerical target. Recruitment and analysis proceeded iteratively, with the researchers assessing the richness, relevance, and diversity of the accounts in relation to the research questions. After approximately 17 interviews, the dataset was considered sufficiently rich to support the development and refinement of the analytic patterns relevant to the study aims. Three additional interviews were conducted to further explore the developing interpretations and to ensure that the dataset provided sufficient depth and variation for the final analysis, resulting in a final sample of 20 participants.

### Researcher–participant relationship

No prior relationship existed between the interviewer and the participants before recruitment. Participants were informed that the interviewer was a public health nurse with training in qualitative research. This disclosure was intended to ensure transparency while minimizing potential power imbalances.

### Data collection

Data were collected through individual in-depth semi-structured interviews using a Personal Information Form and a Semi-Structured Interview Form. The Personal Information Form was developed by the researchers and included questions on participants' sociodemographic and reproductive characteristics, such as age, marital status, parity, and prior gynecological examination experience.

The interview guide was developed through a literature review (2, 9, 10) and refined with input from four experts in public health nursing, midwifery, qualitative research, and communication. It was additionally reviewed by an experienced obstetrician–gynecologist to ensure clinical relevance. It included open-ended questions on women's expectations and emotional responses before the examination, experiences during the procedure, perceptions of communication with healthcare professionals, concerns about privacy and modesty, and reflections after the examination. The guide was pilot tested with two women to assess clarity and relevance, and minor revisions were made before data collection.

All interviews were conducted in a private room in the outpatient gynecology clinic immediately after the vaginal speculum examination, to minimize recall bias and capture immediate emotional and experiential responses. Interviews were conducted by the second researcher, a female public health nurse with prior training and experience in qualitative interviewing.

Interviews lasted approximately 20–45 min, were audio-recorded with participants' permission, and were supplemented by field notes documenting nonverbal expressions and contextual observations. The research team cross-checked transcripts against audio recordings shortly after each interview to ensure accuracy and completeness.

All interviews were conducted and transcribed in Turkish. Data analysis was performed using the original Turkish transcripts. Quotations included in the manuscript were translated into English after completion of the analytic process. Both authors reviewed translated quotations to ensure semantic consistency with the original meanings.

### Data analysis

Analysis followed an iterative and recursive process consistent with the principles of reflexive thematic analysis. First, the transcripts were read repeatedly to become familiar with the data and develop an overall understanding of participants' experiences. Initial ideas and analytic reflections were documented throughout this process. Coding was primarily inductive and data-driven; however, the interview guide and existing literature served as sensitizing concepts during interpretation. Codes were then organized into broader patterns of meaning, and themes were developed and refined through continuous comparison across the dataset.

Theme development was led by the first author, with regular discussions between the two authors to critically examine interpretations and enhance analytic depth. Throughout the analytic process, the researchers engaged in reflexive practice by examining their assumptions, perspectives, and potential influence on the interpretation of the data.

### Trustworthiness

Rigor was ensured through strategies aligned with qualitative research principles. Credibility was supported through prolonged engagement with the data, iterative analysis, and conducting interviews immediately after the examination to minimize recall bias. Analytic rigor was further enhanced through regular team discussions, which facilitated critical reflection on developing interpretations.

Confirmability was addressed by grounding findings in participants' accounts and maintaining an audit trail documenting analytic decisions and theme development. Rather than claiming transferability, we provide contextual descriptions of the setting and participants to allow readers to judge the potential applicability of findings to other contexts. However, the findings should be interpreted within the context of women who completed gynecological examination in a single public hospital setting.

Member checking was not undertaken because interviews were conducted immediately after the clinical encounter and participants were not re-contacted following data collection. Therefore, participants did not review transcripts or provide feedback on the analysis. No repeat interviews were conducted.

### Researcher reflexivity

The researchers recognized that their professional backgrounds, assumptions, and interactions with participants were part of the knowledge-production process and could shape both data collection and interpretation. The second researcher, who conducted the interviews, was a female public health nurse with training and experience in qualitative interviewing. Her clinical background may have influenced the issues that were explored during the interviews, particularly those concerning communication, privacy, emotional safety, and women's perceptions of healthcare professionals.

Rather than assuming that the interviewer could remain completely neutral, the researchers used reflexive practice to examine how their professional positioning and expectations might have shaped the questions asked, the aspects of participants' accounts that received analytic attention, and the development of themes. During the analysis, the first author led the initial coding and theme development, while both authors discussed interpretations and questioned assumptions regarding fear, trust, communication, and vulnerability.

The researchers also considered the potential influence of the clinical setting on participants' accounts. Because interviews were conducted immediately after the examination and within the healthcare environment, participants may have been influenced by their recent care experience or by perceived expectations regarding healthcare professionals. These contextual influences were considered during interpretation rather than treated solely as sources of bias.

Reflexivity was maintained throughout data collection and analysis through ongoing discussion of researchers' assumptions, interpretive decisions, and alternative readings of the data.

The first author, a public health nurse with qualitative research training, conducted the interviews and led the initial coding and theme development. Her clinical background may have influenced the aspects of participants' accounts that received greater analytic attention, particularly regarding communication, privacy, and trust. The second author, also a female public health nurse with experience in qualitative interviewing, contributed to ongoing discussions of interpretations and alternative readings of the data. Throughout the analytic process, both researchers explicitly discussed their assumptions, interpretive decisions, and potential influence on the development of themes.

### Ethical considerations

Ethical approval was obtained from the Ethics Committee of Burdur Mehmet Akif Ersoy University (Approval No: GO 2026/2928; Date: 01 April 2026) prior to data collection. All procedures were conducted in accordance with the ethical standards of the Declaration of Helsinki. Written informed consent was obtained from all participants. Participants were informed about the purpose of the study, the voluntary nature of participation, the confidentiality of their data, and their right to withdraw at any time without any consequences for their care. Participation was entirely voluntary, and refusal to participate or withdrawal from the study did not affect the healthcare services received.

All interviews were conducted in a private setting to ensure confidentiality. Audio recordings and transcripts were anonymized by removing identifying information and assigning participant codes (e.g., P1, P2). All data were securely stored in password-protected digital files accessible only to the research team.

## Results

Twenty women participated in the study. Participants ranged in age from 21 to 62 years. Most participants were married (*n* = 18) and multiparous (*n* = 14). Educational attainment varied, with 10 participants holding a university degree, 6 completing high school, and 4 completing primary school. Body mass index (BMI) ranged from 22.0 to 41.4 kg/m^2^, representing women with normal weight, overweight, and obesity. Four participants reported at least one chronic health condition. The diversity of participants' sociodemographic and reproductive characteristics enabled exploration of a broad range of experiences and perspectives regarding gynecological examination (Table 1). The heterogeneity in age, educational background, parity, and clinical characteristics provided a rich context for exploring how women constructed fear, negotiated vulnerability, and developed trust throughout the gynecological examination experience.

**Table 1:** Sociodemographic, Reproductive, and Clinical Characteristics of the Participants (N = 20).

*P*	Age (years)	Education	Marital Status	Parity (n)	Mode of Delivery	BMI (kg/m^2^)	Chronic Disease
P1	21	University graduate	Married	0	–	23.5	No
P2	23	University graduate	Single	0	–	24.5	No
P3	28	University graduate	Married	0	–	27.3	No
P4	27	University graduate	Married	1	Vaginal	23.2	No
P5	35	High school	Married	1	Cesarean	30.1	No
P6	25	High school	Married	0	–	41.4	Yes
P7	60	Primary school	Married	≥1	Vaginal	23.7	Yes
P8	27	University graduate	Married	1	Vaginal	26.0	No
P9	38	High school	Married	1	Vaginal	26.7	No
P10	23	University graduate	Married	0	–	25.4	No
P11	24	University graduate	Married	1	Vaginal	26.7	No
P12	35	University graduate	Married	1	Cesarean	27.5	No
P13	62	Primary school	Married	≥1	Vaginal	31.2	Yes
P14	32	High school	Married	1	Vaginal	24.8	No
P15	35	University graduate	Married	1	Cesarean	27.5	No
P16	34	High school	Married	1	Cesarean	25.0	No
P17	28	University graduate	Married	1	Vaginal	26.7	No
P18	29	University graduate	Single	0	–	22.0	No
P19	43	High school	Married	1	Cesarean	29.1	Yes
P20	28	University graduate	Married	1	Vaginal	26.0	No

The analysis generated four overarching themes and six interrelated subthemes that collectively illustrate how women construct, negotiate, and reinterpret their experiences of gynecological examination. The four overarching themes were: (1) Constructing Fear Before the Examination, (2) Negotiating Embarrassment and Bodily Exposure, (3) Communication and the Development of Relational Trust, and (4) Reinterpreting the Examination Experience.

Within these overarching themes, six interrelated subthemes were identified: (a) anticipatory fear shaped by social narratives and uncertainty, (b) embarrassment, privacy, and the cultural meaning of bodily exposure, (c) communication as a source of clarity and emotional containment, (d) trust in the clinician as a condition for feeling safe, (e) discrepancy between expected and actual examination experiences, and (f) relief, reappraisal, and greater willingness for future care. Rather than representing discrete categories, these themes depict a dynamic process that unfolds across the examination trajectory. Women entered the clinical encounter with fears and expectations largely shaped by social narratives and uncertainty. During the examination, they negotiated feelings of embarrassment and vulnerability within culturally embedded meanings of bodily exposure and privacy. Communication and supportive interpersonal interactions facilitated the development of relational trust and emotional safety. Finally, many women reinterpreted their initial assumptions in light of their lived experiences, leading to relief, reassurance, and a greater willingness to engage in future gynecological care (Table 2).

**Table 2:** Themes, Subthemes, and the Interpretive Process of Women's Experiences of Gynecological Examination.

Phase of Experience	Overarching Theme	Subthemes	Interpretive Focus
Before the examination	Constructing Fear Before the Examination	Anticipatory fear shaped by social narratives and uncertainty	Fear is often shaped by socially transmitted narratives before direct experience.
During the examination	Negotiating Embarrassment and Bodily Exposure	Embarrassment, privacy, and the cultural meaning of bodily exposure	Vulnerability and bodily exposure are negotiated within sociocultural norms.
During the examination	Communication and the Development of Relational Trust	Communication as a source of clarity and emotional containment; Trust in the clinician as a condition for feeling safe	Communication and trust facilitate emotional safety and reshape the meaning of the examination.
After the examination	Reinterpreting the Examination Experience	Discrepancy between expected and actual experiences; Relief, reappraisal, and greater willingness for future care	Direct experience challenges prior assumptions and promotes positive engagement with future care.

### Theme 1. constructing fear before the examination

#### Subtheme 1.1. anticipatory fear shaped by social narratives and uncertainty

Participants described intense anticipatory anxiety prior to the examination, characterized by fear of pain, fear of diagnosis, and fear of the procedure itself. Notably, these fears were rarely grounded in direct personal experience but were instead shaped through indirect sources of information, particularly narratives circulating within social networks.

Participants frequently referred to stories shared by others as a primary basis for their expectations. These narratives—often emphasizing pain, discomfort, or negative experiences—appeared to function as anticipatory frameworks through which the examination was imagined and emotionally pre-experienced. As one participant stated, “Before coming, I thought it would be very painful. Everyone around me said so” (P5). Similarly, another noted, “I was afraid because of what I had heard, not because of something I experienced” (P11).

These accounts suggest that fear was often shaped through vicarious learning and reinforced by culturally shared narratives. Women entered the clinical setting with preformed expectations that shaped how they anticipated the examination, often amplifying uncertainty and perceived threat. Importantly, several participants later reflected that these expectations had been exaggerated or did not correspond with their actual experiences, suggesting that anticipatory fear was frequently rooted in socially acquired meanings rather than lived experience itself.

### Theme 2. negotiating embarrassment and bodily exposure

#### Subtheme 2.1. embarrassment, privacy, and the cultural meaning of bodily exposure

Concerns related to privacy and modesty were consistently reported and were deeply embedded in culturally shaped understandings of bodily exposure. Participants frequently described feelings of embarrassment associated with positioning and exposing intimate body parts during the examination. These responses were not merely situational reactions but reflected socially and culturally informed expectations regarding modesty and privacy.

Embarrassment appeared to be intensified among women undergoing their first gynecological examination, suggesting that prior socialization and culturally shared meanings of modesty influenced how the examination was anticipated and experienced. In this context, bodily exposure was often interpreted not simply as a clinical necessity but as a socially sensitive and emotionally charged experience.

Environmental conditions within the clinical setting influenced how these feelings were managed. Participants emphasized that privacy-enhancing measures—such as the use of curtains, limiting the number of personnel, and maintaining a controlled environment—contributed to reducing discomfort. As one participant noted, “If there are too many people in the room, I feel more uncomfortable” (P2). Similarly, another stated, “It was difficult for me to relax because I felt exposed” (P4).

Preferences regarding the gender of the clinician were also identified as part of participants' concerns about privacy and emotional comfort. Many women expressed a preference for female physicians, particularly during initial examinations, as this was associated with reduced embarrassment and greater ease in discussing intimate concerns. As one participant noted, “I feel more comfortable with a female doctor because she understands me better” (P9). Another stated, “I prefer a female doctor because I feel less embarrassed” (P1). These preferences appeared to reflect concerns related to modesty, bodily exposure, and emotional comfort rather than technical aspects of care.

Taken together, these findings suggest that gynecological examination is experienced not only as a medical procedure but also as a culturally mediated encounter in which women negotiate feelings of vulnerability, privacy, and bodily exposure.

### Theme 3. communication and the development of relational trust

#### Subtheme 3.1. communication as a source of clarity and emotional containment

Across participants, communication was interpreted as a central mechanism shaping how the examination was understood and emotionally experienced. Uncertainty was consistently identified as a primary source of fear, particularly among women undergoing the examination for the first time. In this context, clear, step-by-step explanations functioned not merely as informational support but as a means of structuring the experience, enabling participants to anticipate what would happen and thereby reducing perceived unpredictability. As one participant explained, “When the doctor explained everything step by step, I felt much calmer because I knew what was going to happen” (P3).

In addition to informational clarity, communication also played a critical role in regulating emotional responses during the examination. Verbal reassurance and ongoing interaction helped shift participants' attention away from discomfort and fostered a sense of being supported during the clinical encounter. As another participant noted, “Talking during the procedure made me feel less nervous; I didn't focus on the discomfort” (P7).

Taken together, these accounts indicate that communication functioned as a relational and interpretive mechanism through which the meaning of the examination was actively reshaped. Rather than simply reducing anxiety, communication facilitated a transition from uncertainty to cognitive clarity and from emotional vulnerability to a more manageable experience. However, this pattern was not universal. A small number of participants reported that, despite receiving explanations, their discomfort and anxiety persisted, suggesting that communication alone may be insufficient to alleviate emotional distress in all circumstances.

#### Subtheme 3.2. trust in the clinician as a condition for tolerating the examination

Beyond communication itself, participants frequently described trust in the clinician as a key condition that enabled them to tolerate the examination despite feelings of vulnerability. Trust was closely linked to perceptions of professional competence, empathy, and respectful interaction. When participants felt listened to, informed, and treated with sensitivity, they reported a greater sense of emotional safety during the examination.

As one participant explained, “I felt safe because I trusted the doctor and knew I was in good hands” (P12). Another participant noted, “The doctor's calm and reassuring manner helped me relax. I trusted her and that made everything easier” (P16).

These accounts suggest that trust was not automatic but was actively developed through interpersonal interactions within the clinical encounter. Trust appeared to function as a relational resource that allowed women to relinquish some of their fears and tolerate the examination despite ongoing discomfort or embarrassment. Nevertheless, trust was not uniformly established. Some participants continued to experience unease even when communication was present, indicating that trust is shaped by a combination of interpersonal, contextual, and individual factors.

### Theme 4. reinterpreting the examination experience

#### Subtheme 4.1. discrepancy between expected and actual experiences

A prominent finding across participants was the discrepancy between anticipated and actual experiences of the examination. Although many participants reported high levels of fear prior to the procedure, the examination itself was frequently described as less painful, less embarrassing, and more manageable than expected. Participants often expressed this contrast explicitly, using phrases such as “It was not as bad as I thought” and “I worried for nothing,” reflecting a clear mismatch between expectation and lived experience.

This discrepancy represented a critical turning point in the experiential process. Pre-existing fears, largely shaped by socially transmitted narratives and uncertainty, were directly challenged by the immediacy of the clinical encounter. As participants moved from anticipation to experience, previously held assumptions were re-evaluated in light of their actual perceptions, thereby weakening fear-based expectations.

Importantly, this shift was not automatic but occurred within the context of supportive communication and the development of relational trust. When participants felt informed, guided, and emotionally supported, the gap between expectations and experience narrowed, facilitating cognitive and emotional recalibration. These findings suggest that direct experience functioned as a mechanism through which socially acquired fears were questioned and reinterpreted.

#### Subtheme 4.2. relief, reappraisal, and greater willingness for future care

Following the examination, many participants reported relief, reassurance, and increased confidence. Several women described feeling “lighter” and more at ease after the procedure, suggesting a release of anticipatory tension. As one participant noted, “After it was over, I felt relieved and more confident about coming again” (P14). Another stated, “Now I am not afraid to come again” (P2).

Importantly, this relief was not merely emotional but also interpretive. Participants described a process of reappraisal in which prior fears and expectations were reconsidered in light of their actual experiences. This reinterpretation contributed to a greater willingness to attend future examinations and, in some cases, to recommend gynecological care to others.

In addition, some participants reported increased awareness of the importance of regular gynecological check-ups, indicating a shift from avoidance toward more proactive engagement with healthcare. Nevertheless, this pattern was not universal. While many women described reduced fear and increased confidence, some participants continued to experience residual discomfort and anxiety. These findings suggest that although the examination may act as a turning point for many women, the reinterpretation of fear and willingness for future care remains shaped by individual experiences and contextual factors***.***

## Discussion

This study suggests that women's experiences of gynecological examination are shaped not only by the procedure itself but also by socially constructed expectations, cultural meanings, and interpersonal experiences during clinical encounters. Participants frequently described entering the examination with fears and expectations derived from second-hand information, informal narratives, and culturally embedded beliefs rather than direct experience. Consistent with previous studies reporting anxiety, embarrassment, and perceived loss of control during gynecological examinations (2, 3, 6, 9, 14), our findings suggest that these emotional responses are often anticipated and socially mediated before the examination itself occurs. The emotional burden of gynecological examination may therefore lie as much in anticipation as in the procedure itself, highlighting the importance of addressing women's expectations before the examination.

Communication and relational trust were interpreted as interconnected mechanisms through which women reinterpreted fear and vulnerability. Participants consistently described clear explanations, step-by-step guidance, and empathetic interactions as essential in reducing uncertainty and promoting emotional safety. This emphasis on communication and trust is also reflected in recent mixed-methods evidence showing that patients value listening, trust, and relationship quality in obstetric and gynecological care (15).

Recent evidence similarly indicates that information, procedural, environmental, and cognitive approaches may influence women's experiences of pelvic examinations, particularly anxiety and discomfort (2, 16). Similarly, women undergoing induction of labor have reported that inadequate information and poor communication contributed to feelings of disempowerment, whereas individualized explanations promoted trust and a greater sense of control (17).

Trust did not arise automatically but developed through respectful, informative, and supportive interactions that enabled women to tolerate the examination despite ongoing feelings of embarrassment or discomfort. These findings align with previous research demonstrating that inadequate communication and insufficient consent undermine trust and contribute to avoidance of future care (8, 9, 16, 18). Our findings further suggest that communication and trust may work together to facilitate the reinterpretation of socially acquired fears within clinical encounters.

Previous qualitative work suggests that women may experience vaginal examinations as routine procedures in which meaningful opportunities to decline are not always perceived (18, 19). This may be particularly relevant when examinations are presented as routine or when information about alternatives and the right to refuse is limited. In the present study, this issue was reflected less in explicit accounts of refusal than in participants' descriptions of how communication, privacy, and interpersonal interactions shaped their sense of control during the examination.

While consent and refusal were not specifically examined in the present study, participants' accounts of communication and emotional safety suggest that women's sense of involvement may be shaped by how the examination is explained and negotiated. Thus, communication should not be understood solely as a strategy for reducing anxiety, but also as part of a respectful interaction that supports informed participation. These findings also resonate with a meta-ethnographic synthesis identifying power dynamics and context of care as important dimensions of women's experiences of vaginal examinations (20). In the present study, these dimensions were reflected less in explicit accounts of refusal and more in participants' descriptions of privacy, communication, clinician behaviors, and their ability to feel safe during the examination.

Concerns related to privacy, modesty, and embarrassment were not merely individual reactions but reflected culturally embedded meanings attached to the body and its exposure. In the Turkish context, where norms surrounding bodily privacy and modesty are strongly emphasized, gynecological examination may represent a temporary disruption of socially regulated boundaries. This interpretation is consistent with previous research indicating that vaginal examinations are experienced within broader cultural frameworks of gender norms and bodily privacy (18, 20, 21).

Recent literature further emphasizes that embarrassment is an important barrier within gynecological care and may influence women's emotional responses, communication with healthcare professionals, and engagement with gynecological services. Butureanu et al. (2026) highlighted the multifaceted clinical impact of patient embarrassment and emphasized the importance of privacy-sensitive, supportive, and patient-centered strategies to mitigate its consequences (22). Consistent with these observations, our findings suggest that embarrassment is not experienced merely as an individual emotional response but is closely connected to perceptions of bodily exposure, privacy, communication, and trust during the examination. Our findings further contribute to this literature by illustrating how embarrassment, privacy, communication, and trust were interconnected across the examination trajectory, suggesting that embarrassment may be experienced not as a fixed emotional response but as something that can be reshaped through the clinical encounter.

Recent qualitative evidence from Türkiye further supports the importance of privacy and perceived bodily control in women's interpretations of gynecological examination. Cambaz Ulaş et al. (2025) reported that Turkish women associated gynecological examination with feelings of shame, worry, pain, and discomfort, highlighting the influence of bodily exposure and the examination context on women's perceptions (14). In the present study, participants similarly emphasized the importance of limiting the number of people present and maintaining privacy during the examination. These findings suggest that privacy may function not only as a physical condition of care but also as an important component of women's perceived control over bodily exposure.

Participants' accounts also pointed to agency and perceived control as important dimensions of the examination experience. Privacy-enhancing measures, the number of people present, and preferences regarding clinician gender were not simply matters of comfort; they shaped women's sense of control over bodily exposure and their ability to feel safe during an intimate procedure. From this perspective, communication may support trust partly by enabling women to anticipate what will happen and to retain a sense of participation in the examination, rather than functioning only as a means of reducing anxiety (10, 18). At the same time, participants did not experience communication and privacy in uniformly positive ways, indicating that a greater sense of control could not be assumed simply from the presence of supportive interactions.

Another notable finding was participants' preference for female clinicians, particularly during initial examinations. This preference appeared to reflect concerns related to modesty, emotional comfort, and perceived understanding rather than technical competence alone. Sensitivity to provider gender preferences, where feasible, may therefore contribute to more patient-centered gynecological care. This finding is also consistent with recent evidence from Türkiye showing that more than half of women preferred a female physician for future gynecological and obstetric examinations, with comfort, privacy, and embarrassment among the main reasons underlying this preference (23). Taken together, these findings suggest that provider gender may function as part of the broader relational and privacy context of gynecological care rather than as an isolated preference.

Finally, the findings suggest that women's experiences of gynecological examination extend beyond the immediate clinical encounter. For many participants, the examination led to relief, increased health awareness, and greater willingness to seek future gynecological care, indicating that early examination experiences may shape long-term engagement with preventive health services. However, this interpretation should be considered cautiously, as participants' willingness to seek future care represented an expressed intention rather than observed subsequent behavior.

Taken together, these findings can be understood as an interpretive pattern in which socially shaped expectations, anticipatory fear, vulnerability, communication, and relational trust are interconnected across the examination experience (Figure 1). Rather than viewing fear, embarrassment, communication, and trust as isolated aspects of the examination experience, our findings illustrate how these experiences may interact across the examination trajectory, from anticipatory fear before the examination to vulnerability and relational trust during the encounter and subsequent reappraisal after the examination. This process-oriented perspective represents the main interpretive contribution of the present study by illustrating how these dimensions were interconnected in participants' accounts. However, the findings do not imply a linear or causal sequence, and women's experiences varied across participants. Some women continued to experience discomfort or anxiety despite supportive communication and reassurance. Therefore, the model should be understood as an interpretive representation of patterns identified in this particular setting rather than as a universal mechanism of how women experience gynecological examination.

**Figure 1:**
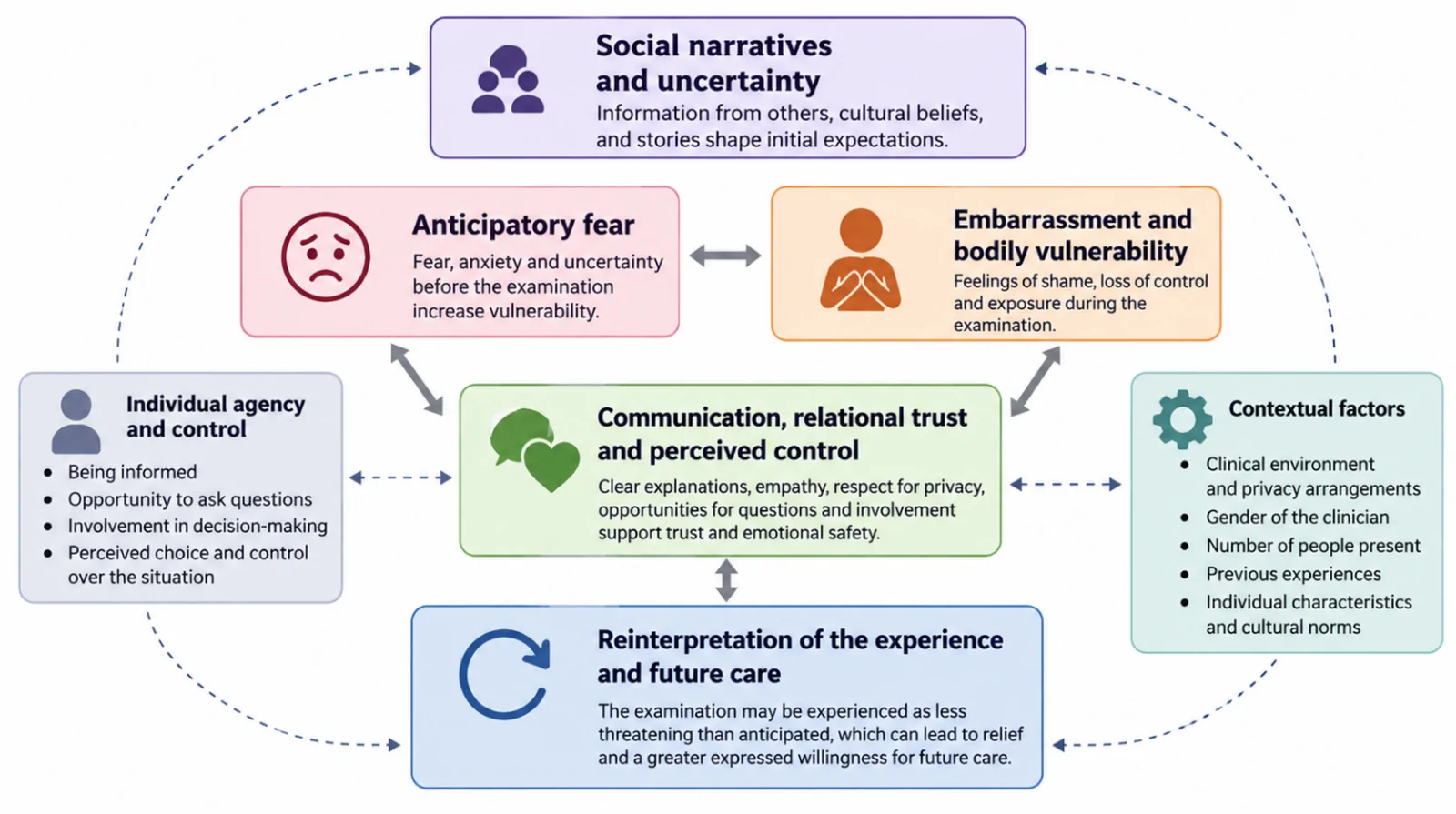
An interpretive process model of women's experiences of gynecological examination. This interpretive model represents patterns identified across participants' accounts rather than a linear causal sequence. The themes are interconnected and may influence one another in various ways. Women's experiences were diverse; some participants continued to experience anxiety or discomfort despite communication and reassurance. Greater willingness for future care represents an expressed intention rather than an observed subsequent behavior.

### Strengths and limitations

This study has several strengths. First, interviews were conducted immediately after the gynecological examination, allowing participants to describe their experiences while emotions and perceptions were still recent and minimizing recall bias. Second, the study employed purposive sampling and an iterative analytic process to capture diverse experiences and develop an in-depth understanding of how women interpret gynecological examinations. Third, the findings provide contextually grounded insights from the Turkish sociocultural setting, where norms related to modesty, privacy, and gender relations may shape women's healthcare experiences in distinctive ways.

The findings should be interpreted within the context of participants who completed the examination and did not experience severe emotional distress. Women with previous traumatic gynecological experiences, sexual trauma histories, or examination avoidance behaviors were not represented. Therefore, the findings should not be interpreted as suggesting that all fear is socially constructed or that anticipatory anxiety outweighs lived traumatic experiences.

Several limitations should be acknowledged. The study was conducted in a single public hospital, which may limit the applicability of findings to other settings. Transferability should therefore be judged by readers in relation to their own context. Because interviews took place shortly after the examination and within the clinical environment, participants may have moderated their responses, increasing the possibility of social desirability bias. In addition, the perspectives of women who avoid or refuse gynecological examinations—arguably those at greatest risk of delayed care—remain unexplored. Finally, although many participants reported increased willingness to seek future care, the study did not examine whether these intentions translated into subsequent healthcare-seeking behaviors.

Importantly, the finding that many women perceived the examination as less distressing than anticipated should not be interpreted to mean that discomfort is solely socially constructed or that communication alone can eliminate distress. Some women experience genuine pain related to conditions such as vulvodynia, vaginismus, pelvic floor dysfunction, or vaginal atrophy. For these individuals, communication remains important but should be complemented by appropriate clinical assessment and management of underlying physical causes of pain. Future research should explore how communication and physical symptom burden interact to shape examination experiences and future healthcare engagement.

## Conclusion

These findings suggest that women's experiences of gynecological examination are shaped by expectations, sociocultural meanings, and the quality of interpersonal interactions during care. Providing clear information, respecting privacy, and fostering trust may improve women's willingness to engage in future gynecological services.

These findings highlight the need to move beyond a purely procedural approach to gynecological care and toward a relational, communication-centered model that prioritizes clear explanations, respect for privacy, empathy, and trust. Future research should examine the experiences of women who avoid or delay gynecological examinations and evaluate whether communication-based interventions lead to sustained improvements in engagement with preventive healthcare.
